# Influence of the nanofibrous morphology on the catalytic activity of NiO nanostructures: an effective impact toward methanol electrooxidation

**DOI:** 10.1186/1556-276X-8-402

**Published:** 2013-09-28

**Authors:** Nasser AM Barakat, Mohammad Ali Abdelkareem, Mohamed El-Newehy, Hak Yong Kim

**Affiliations:** 1 Department of Organic Materials and Fiber Engineering, College of Engineering, Chonbuk National University, Jeonju 561-756, South Korea; 2 Faculty of Engineering, Chemical Engineering Department, Minia University, El Minia 61519, Egypt; 3 Department of Chemistry, College of Science, King Saud University, Riyadh 11451, Saudi Arabia

**Keywords:** Methanol electrooxidation, NiO nanofibers, Electrospinning, Direct methanol fuel cells

## Abstract

In this study, the influence of the morphology on the electrocatalytic activity of nickel oxide nanostructures toward methanol oxidation is investigated. Two nanostructures were utilized: nanoparticles and nanofibers. NiO nanofibers have been synthesized by using the electrospinning technique. Briefly, electrospun nanofiber mats composed of polyvinylpyrolidine and nickel acetate were calcined at 700°C for 1 h. Interestingly, compared to nanoparticles, the nanofibrous morphology strongly enhanced the electrocatalytic performance. The corresponding current densities for the NiO nanofibers and nanoparticles were 25 and 6 mA/cm^2^, respectively. Moreover, the optimum methanol concentration increased to 1 M in case of the nanofibrous morphology while it was 0.1 M for the NiO nanoparticles. Actually, the one-dimensional feature of the nanofibrous morphology facilitates electrons' motion which enhances the electrocatalytic activity. Overall, this study emphasizes the distinct positive impact of the nanofibrous morphology on the electrocatalytic activity which will open a new avenue for modification of the electrocatalysts.

## Background

In the last decades, nanostructural materials have been intensively investigated because of their high surface area which strongly affects their physicochemical characteristics. Of the reported nanostructures shapes, special attention has been paid to the one-dimensional forms such as nanorods, nanowires, and nanofibers. This is due to their potential applications in nanodevices [1-3]. Nanofibers (NFs) received special consideration due to their high axial ratio, good mechanical properties, and easy manageability. Compared to nanoparticles (NPs), nanofibers have small surface area which might have a negative impact upon using them as catalyst in the chemical reactions. However, it was reported that the axial ratio distinctly enhances the catalytic performance, especially in case of electrons' transfer-based processes. For instance, in the photocatalysis, the nanofibrous morphology strongly modifies the performance [3-5].

Direct methanol fuel cells (DMFCs) received much attention during the last decade because methanol is an inexpensive, readily available, and easily stored and transported liquid fuel [6]. DMFCs do not have the fuel storage problem because methanol has a higher energy density than hydrogen - though less than gasoline or diesel fuel. Methanol is also easier to supply to the public using our current infrastructure. In the DMFCs, methanol is directly oxidized to carbon dioxide and water, providing a new way to store and convey the energy [7-9]. The electrocatalysts are the backbone not only of the DMFCs but also of any kind of fuel cells. The successful commercialization is quite dependent on the cost, activity, and durability of the electrocatalysts [9,10]. At present, almost all pre-commercial low-temperature fuel cells use Pt-based electrocatalysts [11-14]. Accordingly, the manufacturing cost is relatively high which constrains wide applications. Moreover, the catalyst poisoning by CO or CHO species is another real problem facing most of the Pt-based electrocatalysts [9,15,16].

To develop new non-precious electrocatalysts, most of the researchers focus only on modifying the composition and ignore the morphology impact. Therefore, many transition metal NPs were introduced as alternative non-precious electrocatalysts to replace the Pt-based materials. However, those NPs suffer from low chemical stability which keeps non-stop research activities to improve the performance as well as the stability.

Compared to metals, it is known that metal oxides have higher chemical stabilities in various media. Accordingly, metal oxides are good candidates as electrocatalysts if the performance could be improved. Recently, NiO nanoparticles deposited on carbon nanotubes showed good behavior toward methanol electrooxidation [17]. In this study, the electrocatalytic activity of NiO toward methanol oxidation could be improved by modification of its nanomorphology. Interestingly, compared to NiO NPs, NiO NFs which were synthesized by the electrospinning process revealed higher performance.

## Main text

### Experimental section

To prepare NiO NFs, a sol–gel composed of nickel acetate tetra-hydrate (NiAc, 1 g, 98% assay Junsei Chemical Co., Ltd, Japan), polyvinylpyrolidine (PVP 1 g, molecular weight = 1,300,000 g/mol, Sigma-Aldrich Corporation, St. Louis, MO, USA) and ethanol (10 g) was electrospun at 10 kV and feeding rate of 0.05 ml/min. The electrospun mat was first vacuously dried and then sintered in air at 700°C. The utilized NiO NPs were synthesized from the same mixture; however, instead of spinning, the solution was dried, grinded and sintered at the same conditions. The electrochemical measurements were performed in a 1 M KOH solution at room temperature. Preparation of the working electrode was carried out by mixing 2 mg of the functional material, 20 μL Nafion solution (5 wt.%) and 400 μL isopropanol. The slurry was sonicated for 30 min at room temperature. Fifteen microliters from the prepared slurry was poured on the active area of the glassy carbon electrode which was then subjected to drying process at 80°C for 20 min. The measurements were performed on VersaSTAT 4 (Oak Ridge, TN, USA) electrochemical analyzer and a conventional three-electrode electrochemical cell. A Pt wire and an Ag/AgCl electrode were used as the auxiliary and reference electrodes, respectively. Surface morphology was studied by scanning electron microscope (SEM; JEOL JSM-5900, JEOL Ltd., Tokyo, Japan) and field-emission scanning electron microscope equipped with EDX analysis tool (FESEM; Hitachi S-7400, Hitachi Ltd., Chiyoda, Tokyo, Japan). Information about the phase and crystallinity was obtained by using Rigaku X-ray diffractometer (XRD, Rigaku Corporation, Tokyo, Japan) with Cu Kα (λ = 1.540 Å) radiation over Bragg angle ranging from 10° to 90°.

## Results and discussion

**Figure 1:**
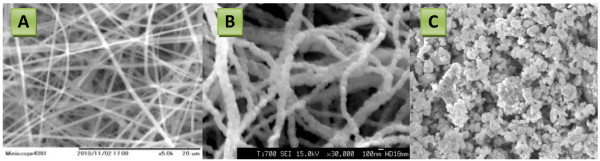
**SEM images of electrospun PVP/NiAc electrospun nanofibers (A), synthesized NiO nanofibers (B), and NPs (C).** SEM images of the electrospun PVP/NiAc nanofiber mats **(A)** and after calcination at 700°C **(B)**. SEM image of the synthesized NiO NPs **(C)**. Scale bar = 200 nm.

The simplicity of the electrospinning process, the diversity of the electrospinnable materials, and the unique features of the obtained electrospun nanofibers provide especial interest for both of the technique and the resultant products. Various polymers have been successfully electrospun into ultrafine fibers in recent years mostly in solvent solution and some in melt form. Moreover, functional inorganic nanofibers can be produced by using sol–gel composed of metal(s) precursor(s) and proper polymer(s). In the field of metallic nanofibers, electrospinning process has a good contribution as it has been invoked to produce several pristine metallic nanofibers [18-21]. Beside the metal alkoxides, metal acetates have been widely utilized as metal precursors, as these promising salts have a good tendency for polycondensation to form electrospinable sol-gels with the proper polymers [22]. The polycondensation reaction can be explained as follows [22]:

where M is Ni. Accordingly, the prepared NiAc/PVP solution produced good morphology, smooth and beads-free electrospun nanofibers, as shown in Figure 1A. Due to the polycondensation characteristic, the calcination of the prepared electrospun nanofibers did not affect the nanofibrous morphology as shown in Figure 1B. Figure 1C represents the SEM image for the synthesized NiO NPs. From Figures 1B and C, it can be concluded that the average diameters of the synthesized NFs and NPs are approximately 70 nm.

**Figure 2:**
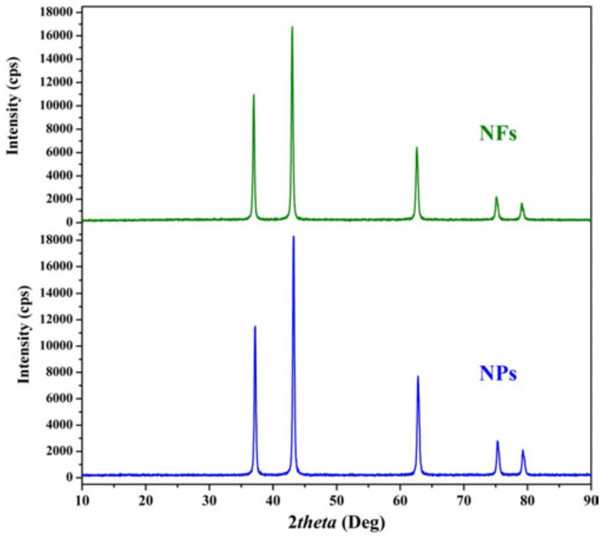
XRD analyses for the prepared NiO nanofibers and nanoparticles.

It was expected that the calcination of the prepared NiAc/PVA nanostructures in air will lead to eliminate the polymer and decompose the metallic precursor to the oxide form; this hypothesis was affirmed by using the XRD analysis. As shown in Figure 2, the XRD spectra of the synthesized NiO NPs and NFs are similar and match the standard spectra of NiO (JCPDS number 44–1159). From the obtained XRD spectra, the grain size could be estimated using Scherrer equation [23]. The determined sizes were 36 and 37 nm for the NPs and NFs, respectively.

**Figure 3:**
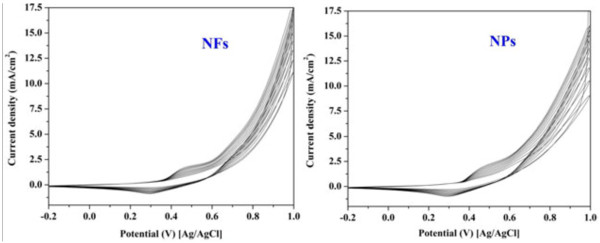
**Consecutive cyclic voltammogram of the synthesized NiO NPs and NFs in 1 M KOH at scan rate of 50 mVs**^**−1**^**.**

Due to its surface oxidation properties, nickel reveals good performance as electrocatalyst. Many materials involving nickel as a component in their manufacture could be used as catalysts in fuel cells. Nickel is commonly used as an electrocatalyst for both anodic and cathodic reactions in organic synthesis, water electrolysis, and electrooxidation of alcohols [24-26]. Surface activation of the nickel-based materials is an important step to create NiOOH compound on the surface and initiate the electrochemical activity. For instance, NiOOH compound has to be originated on the surface to initiate the electrochemical activity. Similarly, the investigated NiO nanostructures in this study were activated by applying cyclic voltages for 50 times in 1 M KOH electrolytes (the utilized scan rate was 100 mV/s). The cyclic voltammetric behaviors of NiO NPs and NFs are shown in Figure 3. In the voltammograms of the nickel oxide nanoparticles and nanofibers, the cathodic and anodic peaks corresponding to Ni(II)/Ni(III) couple are observed at about 0.35 and 0.42 V (vs. Ag/AgCl), respectively. As the chemical composition and the grain size are similar in both nanostructures, the same behavior was obtained as shown in the figure. Typically, these peaks refer to the formation of NiOOH in accordance with these reactions [27-29]:

(2) $$\mathrm{NiO}+H_{2}O\to \mathrm{Ni}{\left(\mathrm{OH}\right)}_{2}$$

(3) $$\mathrm{Ni}{\left(\mathrm{OH}\right)}_{2}+OH^{–}↔\text{NiOOH}+H_{2}O+e$$

Increasing the number of potential sweeps results in a progressive increase of the current density values of the cathodic peak because of the entry of OH^−^ into the surface layer, which leads to the progressive formation of a thicker NiOOH layer corresponding to the NiO/NiOOH transition [24]. It is noteworthy mentioning that the formed NiOOH layer is responsible for the electrocatalytic activity of nickel-based electrocatalysts [17,24].

**Figure 4:**
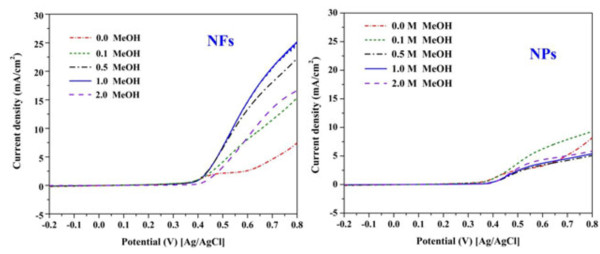
**Linear scan voltammograms for the methanol oxidation on the NiO NPs and NFs surfaces.** Cyclic voltammograms at a scan rate of 50 mVs^−1^ and 25°C for NiO NPs and NFs in 1 M KOH and different methanol concentrations.

The linear scan voltammograms for the methanol oxidation on the NiO NPs and NFs surfaces in different methanol concentrations are shown in Figure 4. The methanol-containing electrolyte was previously purged with argon. The onset potential is an important indicator among the invoked parameters to demonstrate the electrocatalytic activity. The onset potential indicates the electrode overpotential. In other words, the onset potential can be utilized to evaluate the efficacy of the electrocatalyst. In methanol electrooxidation, more negative onset potential indicates high activity and less overpotential. Generally, the main reason behind increasing the onset potential is the OH^−^ and CO adsorbed layer on the surface of the electrodes, this gas layer leads to overpotential [30]. Sometimes, carbon monoxide is an intermediate compound in the methanol electrooxidation; it accumulates on the surface of the electrode until further oxidation step to carbon dioxide occurs. Usually, adsorption of CO appears to take place with the formation of islands of adsorbate [31], and electroactivity appears to be restricted to the outsides of these islands. Accordingly, good catalytic activity is related with the rate of CO removal and/or skipping formation of CO intermediate. From the obtained results, the onset potentials are 0.37 and 0.39 V (vs. Ag/AgCl) for the NiO NPs and NFs, respectively; these values are good compared with many reported materials.

**Figure 5:**
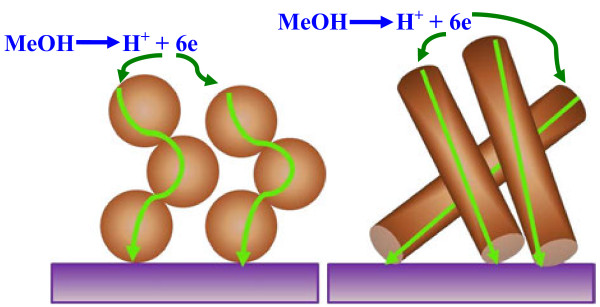
Schematic diagram showing the electron paths in case of NiO nanofibers and nanoparticles.

Two important findings can be observed in Figure 4: First, the nanofibrous morphology strongly enhances the electrocatalytic activity as the maximum current density significantly increased from 6 (in case of NPs) to 25 mA/cm^2^ (in case of NFs). Second, the optimum methanol concentration increased from 0.1 M in case of nanoparticulate morphology to 1 M in case of the nanofibers. Actually, concentrated methanol solution is a target fuel in the DMFCs to reduce the volume. However, increasing of methanol concentration can have a negative influence on the current density, so each electrocatalyst corresponds to a certain methanol concentration. The obtained good performance of the nanofibrous morphology can be assigned to the influence of the one-dimensional feature which facilitates the electron transfer through the electrocatalyst. It is expected that the electron paths through the nanoparticles will be corrugated; however, as the nanofibers have very high axial ratio, almost straight paths are expected. Moreover, within the nanoparticles, the electrons pass through several contact points as they have to move through many nanoparticles; this adds more constraints for the electrons transfer which distinctly affects the catalyst performance. Figure 5 shows a conceptual illustration for the electrons paths through the nanofibrous and nanoparticulate electrocatalysts.

## Conclusions

Electrospinning technique can be utilized to fabricate NiO nanofibers from PVP and nickel acetates sol–gel. The morphology has a distinct influence on the electrocatalytic activity of the nickel oxide nanostructures toward methanol oxidation. Compared to the nanoparticles, the nanofibrous morphology facilitates the electrons' motion which positively affects the performance. It is expected that the good impact of the nanofibrous morphology is a common feature, so it can be utilized with other electrocatalytic materials.

## Competing interests

The authors declare that they have no competing interests.

## Authors’ contributions

NAMB and MAA designed and performed the experimental work and explained the obtained results. NAMB wrote the paper. ME-N and HYK helped in writing of the paper and participated in the experimental work. All authors read and approved the final manuscript.
